# Splenic Abscess in Patients With Prior History of Enteric Fever: A Case Series

**DOI:** 10.7759/cureus.107012

**Published:** 2026-04-14

**Authors:** Shrishti Asani, Navin Kumar, Manoj Kumar, Sandeep Aggarwal

**Affiliations:** 1 Clinical Microbiology, Human Care Medical Charitable Trust, Manipal Hospital, Delhi, IND; 2 Internal Medicine, Human Care Medical Charitable Trust, Manipal Hospital, Delhi, IND; 3 Minimal Access Surgery, Human Care Medical Charitable Trust, Manipal Hospital, Delhi, IND

**Keywords:** antibiotics, enteric fever, pleural effusion, salmonella typhi, splenic abscess

## Abstract

Splenic abscess remains a cause of high morbidity and mortality when not treated on time. We report two cases of splenic abscess who presented with complaints of fever and pain in the abdomen. Both patients were previously diagnosed with enteric fever. The contributory factor for splenic abscess in both cases was non-adherence to antibiotic therapy. Splenic abscesses are caused by a wide range of microorganisms. Blood culture and pus aspirated from the abscess are important to ascertain the microorganisms and their antibiotic susceptibility pattern. Earlier, splenectomy was the mainstay of treatment; however, in our cases, ultrasonography-guided percutaneous aspiration and laparoscopic drainage of abscess with intravenous (IV) antibiotic therapy helped in the complete resolution of the abscess.

## Introduction

Splenic abscess is an uncommon complication of enteric fever in the antibiotic era, with an estimated incidence of 0.14-2% [1]. Several risk factors have been linked to its development, including infective endocarditis, diabetes, trauma, intravenous drug abuse, and hemoglobinopathies [2]. We describe two patients who developed splenic abscesses due to *Salmonella typhi*, each with a prior history of enteric fever but no identifiable risk factors. Prompt diagnosis using imaging modalities such as ultrasonography (USG) or computed tomography (CT), supported by microbiological confirmation, can reduce mortality in affected individuals.

## Case presentation

Case one

A 20-year-old male presented to the emergency department with complaints of high-grade fever and chills for two days. He also reported generalized malaise persisting for one week. The patient had a documented episode of enteric fever one month earlier, for which he visited a local practitioner and received ampicillin-sulbactam. The dose, duration, and compliance of antibiotic treatment were unclear. He showed partial improvement with temporary resolution of fever, followed by a relapse of symptoms. There was no history of immunosuppressive conditions or medications. He was admitted for further evaluation and clinical management.

At presentation, the patient was febrile (102°F) with a pulse rate of 120 beats per minute, blood pressure of 114/76 mm Hg, and respiratory rate of 20 breaths per minute. General examination revealed no pallor, icterus, or cyanosis. There were no signs of meningeal irritation.

On abdominal examination, mild tenderness was noted in the left hypochondrium without guarding or rebound tenderness. No hepatosplenomegaly was observed on palpation. The cardiovascular and respiratory systems were within normal limits.

Initial laboratory tests revealed leukocytosis and an elevated C-reactive protein (CRP). Liver function tests showed mild elevation of aspartate aminotransferase and alanine aminotransferase. Dengue NS1 antigen and peripheral smear for malaria parasites were negative. Blood cultures remained sterile after 48 hours of aerobic incubation. Stool culture showed no growth of *Salmonella*,* Shigella*,or *Vibrio* species. Viral serology tests for human immunodeficiency virus (HIV), hepatitis B virus (HBV), and hepatitis C virus (HCV) were non-reactive. Hemoglobin electrophoresis was done to rule out hemoglobinopathies, including sickle cell disease, and revealed a normal pattern.

**Figure 1:**
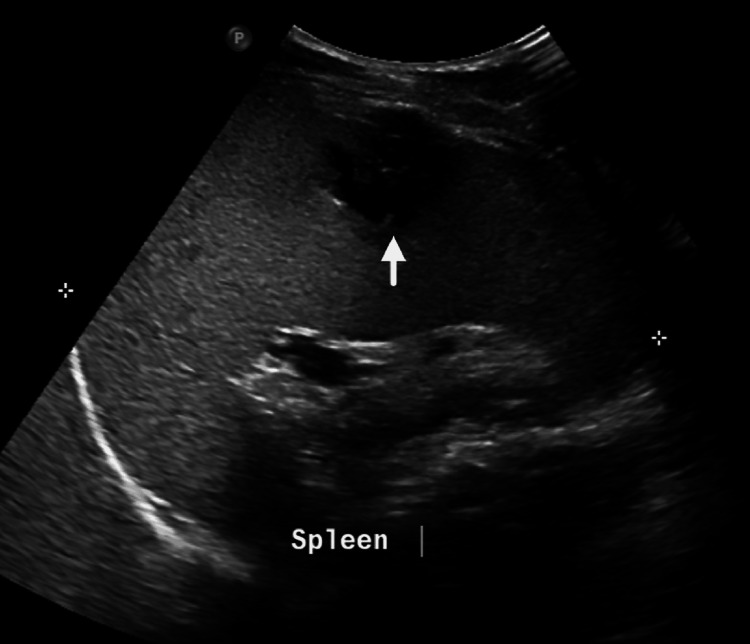
USG abdomen showing mild splenomegaly with a solitary heterogenous hypoechoic lesion suggestive of an abscess USG: ultrasonography.

On abdominal USG, mild splenomegaly with a solitary heterogeneous hypoechoic lesion in the spleen measuring approximately 2.5 × 2.1 × 2 cm with internal fluid collection (volume ~5.6 cc) suggestive of an abscess was seen (Figure 1).

Ultrasound-guided percutaneous drainage of the splenic abscess was performed. Pus collected was sent for aerobic culture, and GeneXpert (Cepheid, Sunnydale, USA) for *Mycobacterium tuberculosis *(MTB). He was empirically started on intravenous (IV) ceftriaxone. Culture grew *Salmonella typhi*, sensitive to ceftriaxone, azithromycin, cefixime, trimethoprim-sulfamethoxazole, and meropenem, but resistant to ciprofloxacin.

Diagnosis of complicated enteric fever with splenic abscess was confirmed. The patient showed clinical improvement within five days of IV ceftriaxone and was discharged in stable condition to complete a 14-day course of antibiotics. At the six-week follow-up, USG abdomen revealed complete resolution of the abscess.

Case two

A 34-year-old male presented with a one-month history of high-grade fever associated with chills. He was diagnosed with typhoid fever one month back based on a positive Widal test and was started on oral antibiotics. The dose and duration of the antibiotic treatment were not available. Although he initially showed some improvement, he later on developed abdominal pain.

At presentation, the patient was febrile (100 °F) with a pulse rate of 90 beats per minute, blood pressure of 118/74 mm Hg, and respiratory rate of 20 breaths per minute. His examination also revealed no pallor, icterus, or cyanosis.

On systemic examination, abdominal assessment revealed severe tenderness with guarding in the left hypochondrium. Respiratory examination showed decreased air entry in the left lower lung, while the cardiovascular and central nervous system examinations were within normal limits.

Laboratory tests showed a total leukocyte count of 12,750 cells/mm³. Liver function tests showed mildly elevated alkaline phosphatase. Blood culture remained sterile after 48 hours of aerobic incubation, and serology tests for HIV, HBV, and HCV were non-reactive.

**Figure 2:**
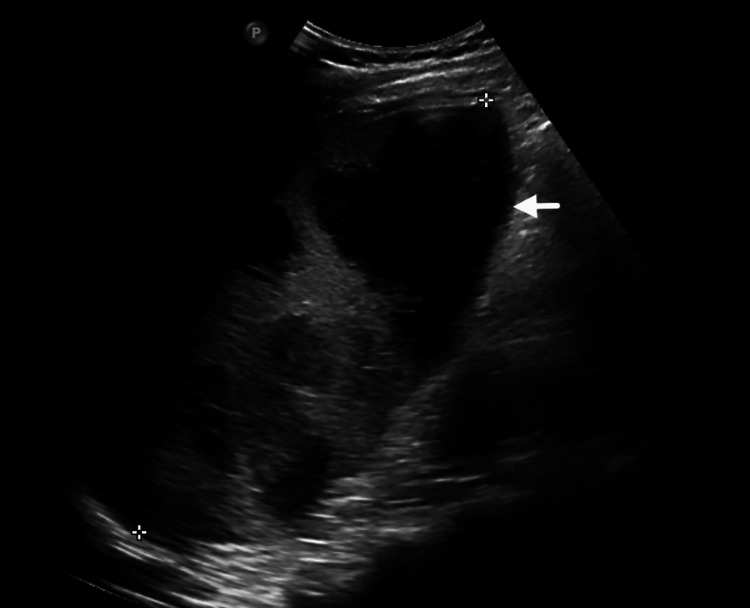
USG abdomen showing splenomegaly with a large, lobulated and partially liquefied abscess USG: ultrasonography.

Abdominal USG revealed splenomegaly (16.7 cm) with a lobulated, partially liquefied abscess measuring approximately 300 cc (Figure 2). Contrast-enhanced CT of the abdomen confirmed splenomegaly with multiple abscesses, the largest measuring 10 cm. Thoracic USG demonstrated left pleural effusion (34 cc) with subsegmental collapse/consolidation. Chest radiography showed left lower zone opacity with silhouetting of the costophrenic angle, consistent with pleural effusion and underlying collapse/consolidation.

**Figure 3:**
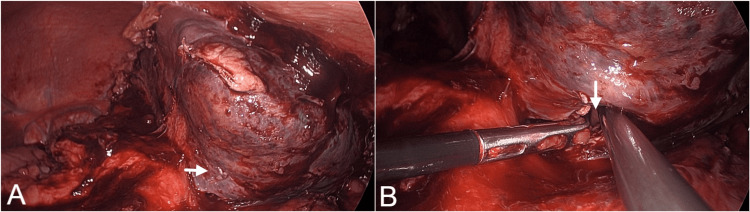
Laparoscopic drainage of splenic abscess (A) and (B) Intraoperative images of splenic abscess showing site of splenic abscess and abscess cavity after pus drainage

The patient was started empirically on IV ceftriaxone and metronidazole and underwent laparoscopic drainage of abscesses (Figure 3). Pus aspirated was sent for aerobic culture and GeneXpert for MTB. Culture grew *Salmonella typhi,* which was susceptible to ceftriaxone, azithromycin, cefixime, trimethoprim-sulfamethoxazole, and meropenem, but resistant to ciprofloxacin. GeneXpert was negative for MTB.

Patient improved clinically. He was discharged on oral cefixime and azithromycin, with advice to follow up after seven days for USG and chest radiography.

Four days after discharge, the patient returned with complaints of intermittent cough and dyspnea. On examination, decreased air entry was noted over the left lower lung fields. Repeat chest radiography revealed homogeneous opacification of the left mid and lower lung zones, suggestive of pleural effusion. Pleural fluid aspiration yielded 1,100 ml of pleural fluid. Analysis of the pleural fluid showed a total cell count of 2,770 cells/mm³ with 88% lymphocytes and 12% neutrophils, and a protein concentration of 5.5 g/dL. The serum protein concentration was 6.7 g/dL. The pleural fluid to serum protein ratio of 0.82 was consistent with exudative effusion as per Light's criteria. Both pleural fluid culture and repeat blood culture were sterile.

The patient was re-admitted and started on IV meropenem. He showed significant clinical improvement within 72 hours and completed a seven-day course of IV meropenem. He was then discharged in stable condition on oral cefixime and azithromycin for an additional 14 days. A combination of cefixime and azithromycin was given, targeting *Salmonella typhi *through both extracellular and intracellular activity. This dual therapy ensured complete eradication of the infection and reduced the risk of relapse. At the two-week follow-up, the patient remained clinically stable, and repeat abdominal USG demonstrated complete resolution of the splenic abscesses.

## Discussion

Splenic abscesses are unusual, and diagnosis is based on clinical history, physical examination, and radiological findings. In our series, both patients presented with fever and left upper-quadrant pain. One of the two patient also had pleural effusion (50%). The clinical features observed were consistent with previous studies. Lee et al. reported fever in 68.7 %, left upper quadrant pain in 37.5%, and left-sided pleural effusions in 50 % of cases [3].

Our first case presented with one week of symptoms, while second case had symptoms for past one-month. Various studies have shown variation in the mean duration of symptoms at the time of presentation which was 3.1 days as per a study by Farooque et al. to one-month as per a study by Divyashree et al. [2,4]. Case one had a solitary splenic abscess whereas CT abdomen of case two showed multiple abscesses. Farooque et al. reported that solitary abscesses are more common [4]. Whereas several other studies reported multiple splenic abscesses being more common than solitary abscess [5,6].

Both our patients had a preceding history of typhoid fever, and the diagnosis of splenic abscess was confirmed by radiological findings and microbiologically from aspirated pus. Some reports noted difficulty in eliciting a history of recent illness in their patients, and cases of Kaur et al. were clinically misdiagnosed as pancreatitis due to overlapping symptoms and elevated pancreatic enzymes [1]. In our patients, microbiological confirmation was established through culture of aspirated pus, while blood cultures remained sterile, similar to findings by Divyashree et al. [2]. This contrasts with other reports that describe up to half of splenic abscess cases showing positive blood cultures [2]. In both cases, the pus culture grew *Salmonella typhi*. Ganesan et al. reviewed 33 cases of splenic abscess caused by *Salmonella* species, *Salmonella typhi,* and *Salmonella paratyphi A* were reported in 29 and four cases, respectively [5].

Spleen being a reticuloendothelial organ, has phagocytic activity, and leukocytes prevent the occurrence of splenic abscess [7]. Splenic abscess typically develops following splenic injury due to infarction or trauma, with commonest being systemic bacteremia due to infective endocarditis. In our cases, the abscesses were most likely the result of hematogenous seeding secondary to bacteremia following *Salmonella typhi *infection, comparable to the case described by Gupta et al. [8].

## Conclusions

Although splenic abscess caused by *Salmonella typhi *is rare, this must be kept in mind especially in low and middle-income countries where the incidence of enteric fever is high. Early diagnosis and prompt treatment can help in preventing further complications.
